# EpCAM aptamer mediated cancer cell specific delivery of EpCAM siRNA using polymeric nanocomplex

**DOI:** 10.1186/s12929-014-0108-9

**Published:** 2015-01-09

**Authors:** Nithya Subramanian, Jagat R Kanwar, Prasanna kumar Athalya, Narayanan Janakiraman, Vikas Khetan, Rupinder K Kanwar, Sailaja Eluchuri, Subramanian Krishnakumar

**Affiliations:** 1 Department of Nanobiotechnology, Vision Research Foundation, Kamalnayan Bajaj Institute for Research in Vision and Ophthalmology, 18 College Road, Chennai, 600006 Tamil Nadu India; 2 Nanomedicine Laboratory of Immunology and Molecular Biomedical Research (LIMBR), School of Medicine (SoM), Molecular and Medical Research (MMR) Strategic Research Centre, Faculty of Health, Deakin University, Geelong, Victoria 3217 Australia; 3 L & T Ocular Pathology department, Vision Research Foundation, Kamalnayan Bajaj Institute for Research in Vision and Ophthalmology, Chennai, India; 4 Departments of Ocular Oncology and Vitreoretina, Medical Research Foundation, Sankara Nethralaya Chennai, India

**Keywords:** EpCAM, Aptamer, PEI-EpApt-SiEp, siRNA delivery, Cancer targeting

## Abstract

**Background:**

Epithelial cell adhesion molecule (EpCAM) is overexpressed in solid tumors and regarded as a putative cancer stem cell marker. Here, we report that employing EpCAM aptamer (EpApt) and EpCAM siRNA (SiEp) dual approach, for the targeted delivery of siRNA to EpCAM positive cancer cells, efficiently inhibits cancer cell proliferation.

**Results:**

Targeted delivery of siRNA using polyethyleneimine is one of the efficient methods for gene delivery, and thus, we developed a novel aptamer-PEI-siRNA nanocomplex for EpCAM targeting. PEI nanocomplex synthesized with EpCAM aptamer (EpApt) and EpCAM siRNA (SiEp) showed 198 nm diameter sized particles by dynamic light scattering, spherical shaped particles, of 151 ± 11 nm size by TEM. The surface charge of the nanoparticles was −30.0 mV using zeta potential measurements. Gel retardation assay confirmed the PEI-EpApt-SiEp nanoparticles formation. The difference in size observed by DLS and TEM could be due to coating of aptamer and siRNA on PEI nanocore. Flow cytometry analysis revealed that PEI-EpApt-SiEp has superior binding to cancer cells compared to EpApt or scramble aptamer (ScrApt) or PEI-ScrApt-SiEp. PEI-EpApt-SiEp downregulated EpCAM and inhibited selectively the cell proliferation of MCF-7 and WERI-Rb1 cells.

**Conclusions:**

The PEI nanocomplex fabricated with EpApt and siEp was able to target EpCAM tumor cells, deliver the siRNA and silence the target gene. This nanocomplex exhibited decreased cell proliferation than the scrambled aptamer loaded nanocomplex in the EpCAM expressing cancer cells and may have potential for EpCAM targeting *in vivo*.

**Electronic supplementary material:**

The online version of this article (doi:10.1186/s12929-014-0108-9) contains supplementary material, which is available to authorized users.

## Background

Epithelial cell adhesion molecule (EpCAM) is highly expressed in most of the solid tumors. It has been reported as a putative cancer stem cell marker [1,2], and is regarded as a target antigen for cancer therapies using antibody, ankyrins and aptamers [3,4]. Since an aptamer based tumor targeting can rescue the inherent issues associated with antibody, such as larger size, immunogenicity, both DNA and RNA aptamers against EpCAM were developed using Systemic Evolution of Ligands by Exponential Algorithm (SELEX) technology, and these aptamers can be potentially utilized for therapeutic purpose [5,6]. EpCAM RNA aptamer functionalized with either doxorubicin or SPION-nucleolin aptamers, or bovine lactoferrin showed greater specificity for cancer cells [7,8]. The EpCAM RNA aptamer was used to deliver curcumin using PLGA lecithin nanoparticles against colorectal adenocarcinoma cells [9].

Even though there are various strategies for targeting cancer cells, RNA interference (RNAi) is still regarded as the more suitable approach [10]. RNAi based therapy is preferred over antibody based strategy due to less immunogenicity, easy production and target-specific gene silencing. Clinical trials using siRNA against ribonucleotide reductase subunit M2 (RRM2) studies show promising results in humans [11]. Nanocarrier based delivery is generally preferred due to lack of high penetrance of siRNA into the cancer cells [12]. In this regard, polymer based nanocarriers show less immunogenicity, toxicity, and has better nucleic acid delivery compared to the viral carriers. Polymeric nanocarrier such as polyethyleneimine [13] is one of the most explored cationic carriers due to its high transfection efficiency [14]. However, virosome and exosome based nanocarriers for the delivery of siRNAs has also been reported earlier [15-17]. Cell penetrating peptides and affibodies that can deliver siRNA are as well reported [18,19]. Among these nanocarriers, PEI has the best properties for the condensation of nucleic acids into a nanosized complex. The positively charged PEI polymer, complexes with negatively charged oligonucleotide through electrostatic interactions and forms a stable nanocomplex even in the presence of the serum [20-22]. Also PEI nanocarrier was reported for their non-mutagenic property and not to induce inflammatory response [23,24].

PEI nanocomplex has been efficiently used for the delivery of siRNA using target specific antibody or affibody or aptamer. When used to deliver, nucleic acid aptamers (PSMA, Sgc8c and Muc1) fabricated with PEI-siRNA, target specific delivery to cancer cells was observed [25-27]. Similarly, the gold nanoparticles fabricated with SiEp and EpCAM antibody, showed targeted silencing of EpCAM in the RB cell line [28]. In the present study, we utilized an aptamer based strategy to deliver siRNA against EpCAM as a PEI based nanoformulation. The charge based stabilization of the PEI with the sodium citrate is found to be more efficient for loading the siRNA and the aptamer for a targeted delivery [29]. Therefore, we targeted the EpCAM expressing cancer cells, using an EpCAM aptamer and SiEp loaded PEI nanocomplex, stabilized with sodium citrate for silencing EpCAM that can result in the inhibition of the cancer cell proliferation.

## Methods

### Cell culture

Breast cancer cell line (MCF-7) and retinoblastoma cell line (WERI-Rb1) were obtained from Riken cell bank, Japan and were grown in DMEM and RPMI 1640 (Sigma-Aldrich, Bangalore, India) supplemented with 10% FBS (fetal bovine serum) (Invitrogen, Bangalore, India) at 37°C and 5% CO_2_ humidified atmosphere. 1× antibiotic and antimycotic solution (Himedia, Mumbai, India) was added to the complete growth media used for cell culture.

### Synthesis and stabilization of PEI: sodium citrate nanocomplex

PEI stock solution (100 μg/mL) was prepared and the pH was adjusted to 6.0 using 1 N HCl. The charge ratios of 1:1, 1:1.5, 1:3, 1:5 and 1:7 were prepared between PEI and sodium citrate based on the amine to the carboxylic groups present in the complexes [29]. The reaction was incubated for 10 min in a reaction volume of 1 mL. The size and the zeta-potential of the nanocomplexes were measured using Zetasizer Nano ZS (Malvern). The ratio 1:1.5 of PEI to sodium citrate was used for studying the cytotoxicity in the cell lines, and also for synthesizing the PEI-EpApt-SiEp and PEI-ScrApt-SiEp nanocomplex.

### Fabrication and characterization of PEI-Aptamer-siRNA nanocomplex

The PEI concentration (0.3 μg/mL) that was determined as nontoxic to the cells, was chosen and complexed with EpCAM Aptamer (EpApt) and EpCAM siRNA (SiEp). Different concentrations of EpApt (100 nM to 300 nM) and SiEp (100 nM to 300 nM) were added to the PEI, and incubated at room temperature for 10 min. The PEI alone and PEI-Aptamer-siRNA (PEI-Apt-siRNA) nanocomplexes were electrophoresed on 2% agarose gel in TAE buffer to confirm the complex formation. The size and zeta potential of the nanocomplex saturated with aptamer and siRNA were measured using Zetasizer. The size of PEI nanocomplex was tested in serum supplemented and serum free media using zetasizer. TEM was performed for the PEI-Apt-siRNA nanocomplex (200 nM EpApt and 200 nM siRNA) and imaged at 80 V (TEM, Philips, CM12 STEM, Netherlands).

### Cellular uptake of the PEI-Aptamer siRNA nanocomplex

Cellular uptake of the PEI-Apt-siRNA nanocomplex was performed in MCF-7 and WERI-Rb1 cells using flow cytometry (BD Science FACS Caliber). The 200 nM of aptamer alone or PEI-Apt-siRNA nanocomplex (200 nM EpApt and 200 nM siRNA) were incubated with 2 × 10^5^ cells for 4 h followed by washing with 1× PBS two times and the cells were analyzed using BD FACS Calibur. The unstained cells and the scrambled aptamer treated cells were included as controls. The uptake of the aptamer alone, and PEI-Apt-siRNA nanocomplex was visualized using fluorescent Axio Observer microscopy (Zeiss, Germany).

### Silencing and cytotoxic effect of PEI aptamer-siRNA nanocomplex on cell culture

The effect of siRNA delivery to the cells was evaluated using real time PCR or quantitative (qPCR) and Western blotting. MCF-7 and WERI-Rb1 cells were seeded at a density of 2 × 10^5^ cells per well of 6 well plate. After 24 h of seeding, the PEI alone or PEI-Apt-siRNA nanocomplexes or lipofectamine-SiEp were added to the cells in serum deprived media and incubated further for 4 h, followed by the addition of serum containing media. The nanocomplex treated cells were incubated for 48 h to study the specific delivery of the EpCAM siRNA to the targeted cells. The total RNA from treated cells was extracted using Tri reagent (Sigma, Bangalore, India) and the cDNA was synthesized using Verso cDNA synthesis kit. The levels of EpCAM mRNA expression were measured in MCF-7 and WERI-Rb1 cell lines using TaqMan real time PCR reagents (Applied biosystems, Foster city, USA) using GAPDH expression as an internal control. The immunoblot analysis was performed to check the EpCAM protein expression in both cell lines treated with the PEI-Apt-siRNA nanocomplexes or lipofectamine-SiEp. For studying cytotoxic effect of these complexes, 7,500 cells per well in 96 well plate were seeded, and treated as per the details provided in the Additional file 1.

### Statistical analysis

Statistical analysis was performed using unpaired student t-test for all experiments, except for the cell viability assay, analysis of variance (one-way) is performed and data interpreted. The results are mean of three independent experiments. Each experiment consisted of 3 replicates and was performed atleast twice or thrice. P values less than 0.001 were considered very significant and indicated with “**” and P ≤ 0.05 is considered as significant and indicated with “*”.

## Results

### Synthesis and characterization of PEI nanocomplex with EpCAM Aptamer and siRNA

**Figure 1:**
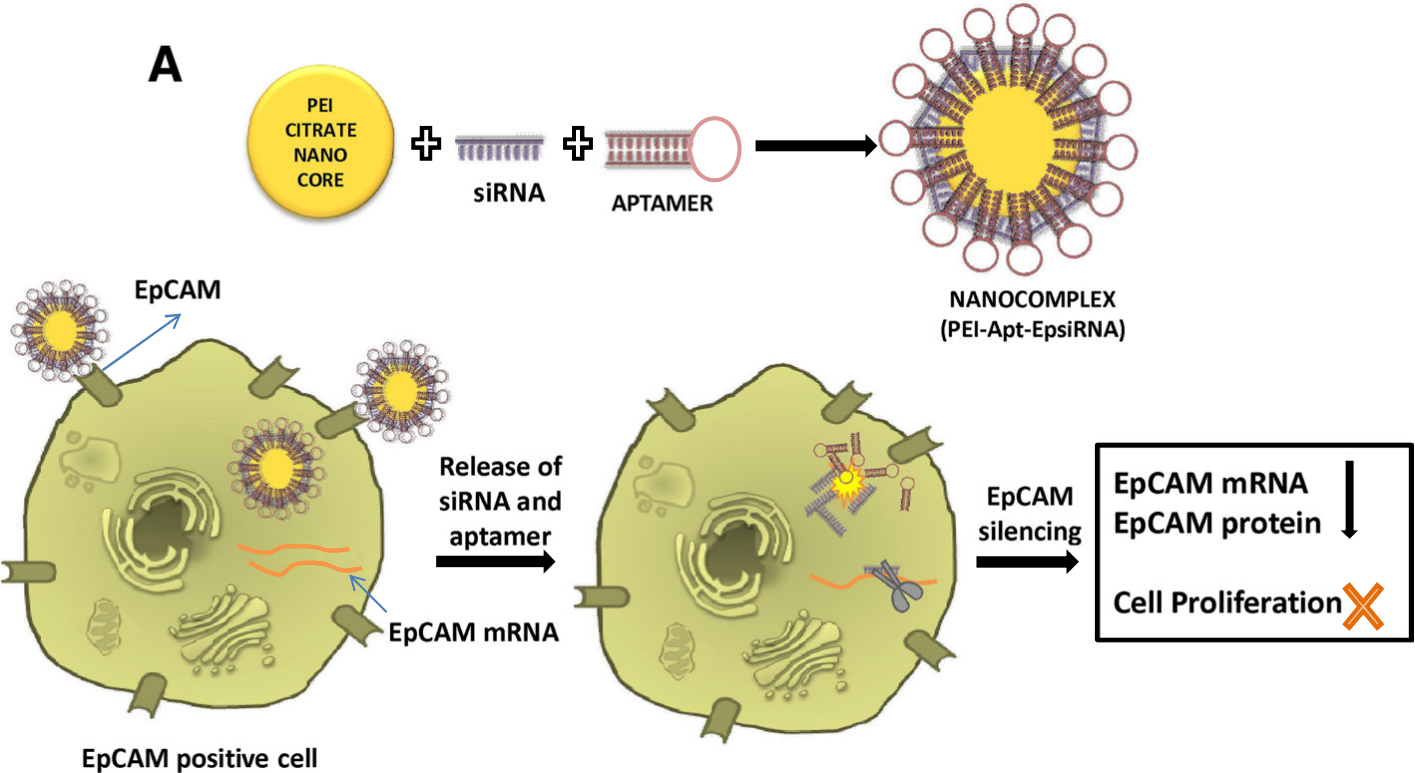
**Schematic showing the cell specific silencing strategy mediated by PEI nanocomplex fabricated with aptamer and siRNA.** PEI nanoparticle is formed using sodium citrate as charge stabilizer, followed by the addition of siRNA and EpCAM aptamer to form the PEI-Apt-siRNA complex. This complex guided by the aptamer, binds to the EpCAM positive cells and delivers the siRNA in the cytoplasm resulting in target gene silencing and inhibition of cellular function pertained to it.

**Figure 2:**
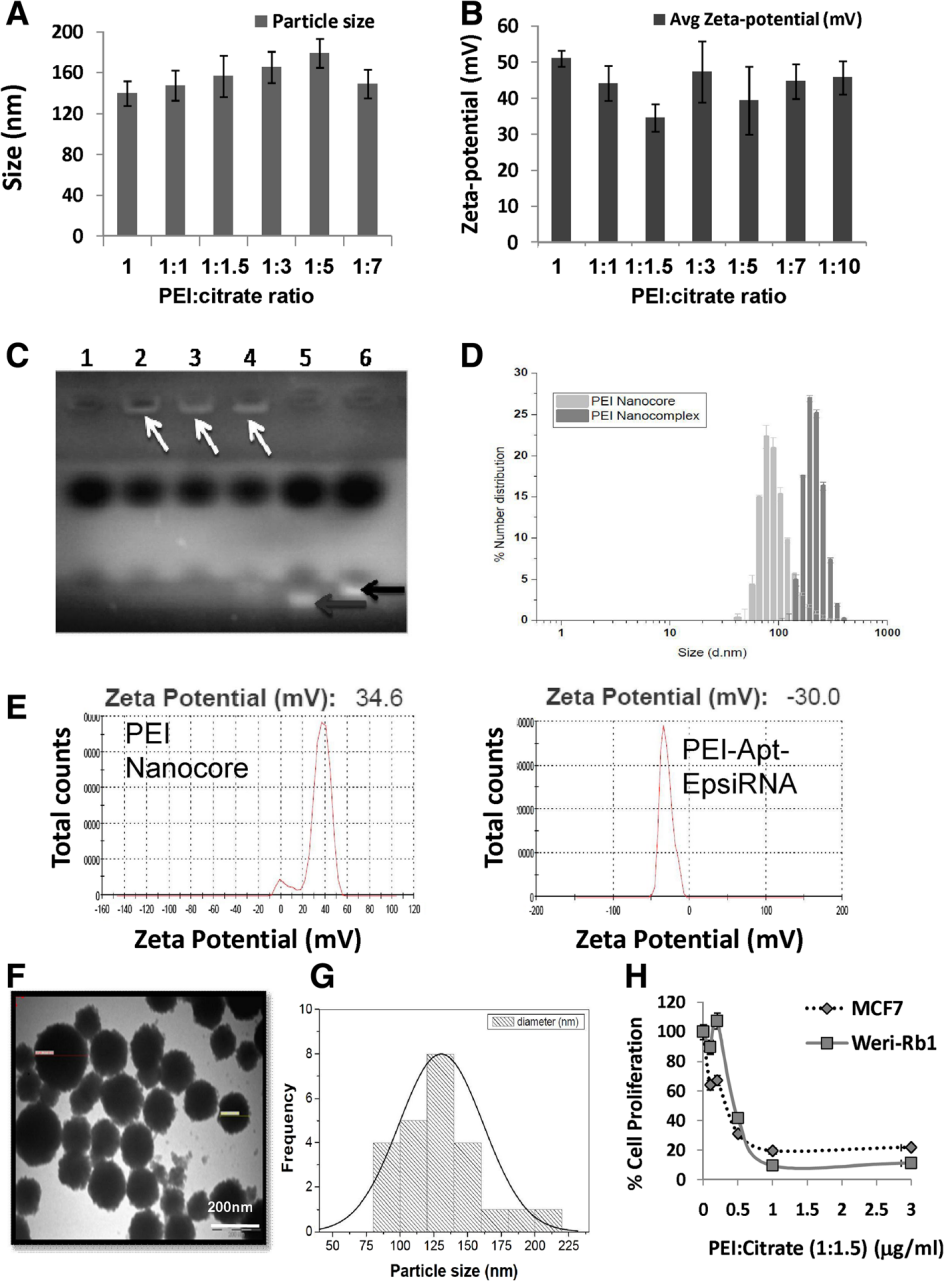
**Effect of citrate on the nanocomplex size, charge and characterization of the nanocomplex.** Graph showing the hydrodynamic sizes **(A)** and surface charge **(B)** of PEI: citrate nanocomplexes formed using different ratio of PEI to citrate measured using zetasizer. **C**. Titration of different concentration of aptamer and siRNA was carried out and loaded onto 2% agarose gel with ethidium bromide and checked for the retention of the PEI complex on the wells. Lane 3 shows 200nM of aptamer and 200nM of siRNA is required to saturate 0.3μgs of PEI and the next highest concentration of 300nM of aptamer & siRNA respectively had some amount of free siRNA and aptamer (lane 4). Lane 5 & 6 indicates free aptamer and siRNA indicated with red and black arrow respectively. On the right, histogram plot showing Particle size distribution of the PEI-Apt-siRNA nanocomplex. **D**. Histogram overlay plot showing the percent number distribution of the PEI nanocore alone and PEI-nanocomplex with aptamer and siRNA (hydrodynamic diameter in nm) measured using zetasizer. **E**. Graph showing the total counts of representative zeta-potential (mV) of the PEI nanocore and the PEI-Apt-siEp nanocomplex. **F**. TEM images of the PEI nanocomplex left panel showing the uniformity of particle distribution and histogram showing distinct particles with a spherical shape **(G)**. **H**. Graph showing the percentage cell proliferation upon treating with different concentration from 0.1 to 3 μg/mL of PEI on MCF-7 and WERI-Rb1 cell line till 48 h. Inhibitory effect of PEI on the cell proliferation and mitrochondrial activity was assessed by MTT assay.

We have synthesized PEI nanocore and PEI-Apt-SiEp nanocomplex as shown in schematic representation (Figure 1). The illustration describes the process of PEI nanocomplex synthesis followed by siRNA and aptamer addition. We tested the hypothesis that the nanocomplex when added to cells, would specifically bind to the EpCAM receptor on the membrane, and would release the aptamers, and siRNA into the cytoplasm, leading to the silencing of EpCAM mRNA. PEI nanocore was prepared by stabilizing its charge using sodium citrate to form an optimal core in aqueous solution. The size of the PEI nanocore depended on the ratios of citrate to PEI i.e., carboxyl group charge/amine group charge ratio (R). The PEI: citrate nanocore synthesized with different R ratios showed particles sizes ranging from 75–250 nm and zeta-potential ranging from 34 mV to 48 mV (positively charged). The ratio 1:1.5 of PEI: citrate resulted in optimum size of 156 ± 6.8 nm and zeta potential of 34.6 mV (Figure 2A & B). The nanocomplex formation was mediated by stabilization of the positively-charged PEI by negatively charged sodium citrate, and further by the electrostatic interaction between the nanocore, the siRNA and the aptamer. The PEI-Apt-siRNA nanocomplexes were synthesized in aqueous media with varying amounts of the aptamer and siRNA. To the PEI nanocore, siRNA was added first followed by aptamer. The aptamer was added finally to form the complex as it enables the complex to recognize the EpCAM on the cell surface. The addition of the aptamer and the siRNA together would lead to a lesser occupancy of aptamer on the particle surface thereby leading to lower binding efficiency of the particles; hence the stepwise complex formation was maintained. The synthesized complexes exhibited retardation on an agarose gel (Figure 2C). We observed that 200 nM of aptamer and siRNA, were able to saturate the PEI: citrate nanocore beyond which free aptamer and siRNA were present, in addition to that the complex retained in the well (Figure 2C, lane 4). Therefore, 200 nM of EpApt and 200 nM SiEp complexed with PEI-citrate (PEI-EpApt-SiEp) nanocomplex was used for further studies. This nanocomplex exhibited a hydrodynamic diameter of 198 ± 14.2 nm and zeta potential of −30.0 mV. The percent number distribution of the sizes of PEI nanocore alone and nanocomplex are shown in Figure 2D. Figure 2E shows the zeta potential of the PEI nanocore alone and nanocomplex, respectively. There was a complete shift in the surface charge(34.6mV) due to the aptamer and siRNA addition leading to negative surface charge(−30.0). The TEM analysis exhibited a particle size of 151 ± 11 nm. The TEM analysis of both PEI and PEI-Apt-siRNA nanocomplexes showed spherical particles (Figure 2F). The frequency of sizes of the particles as observed by TEM is shown as histogram (Figure 2G).

For the future *in vivo* applications, we additionally studied the effect of serum on the size and the charge of the PEI nanoparticles prepared in aqueous media. For this, we added the prepared complexes to the RPMI media with and without the serum. The size of the nanocomplex in media with and without serum are plotted as overlay percent number distribution and exhibited very minimal difference (Additional file 2: Figure S1A) and the charge of the nanocomplex incubated in media with and without serum showed minor changes were found to be -18 mV and −18.7 mV respectively (Additional file 2: Figure S1B & C).

### Cytotoxic effect of PEI polymer on cells

MCF-7 and WERI-Rb1 were used to study the cytotoxic effect of the PEI on cells. The cytotoxicity of PEI was found to be lesser with decreasing concentration of the PEI i.e., 3 μg/mL concentration showed higher toxicity, 0.3 μg/mL and 0.1 μg/mL showed lesser cytotoxicity, hence 0.3 μg/mL was chosen to rule out any non-specific cellular effects that can be attributed by PEI (Figure 2E). Therefore, PEI nanocomplexes for the aptamer and the siRNA functionalization were carried out with the concentration of 0.3 μg/mL of PEI.

### Cellular uptake of PEI Aptamer siRNA complex

**Figure 3:**
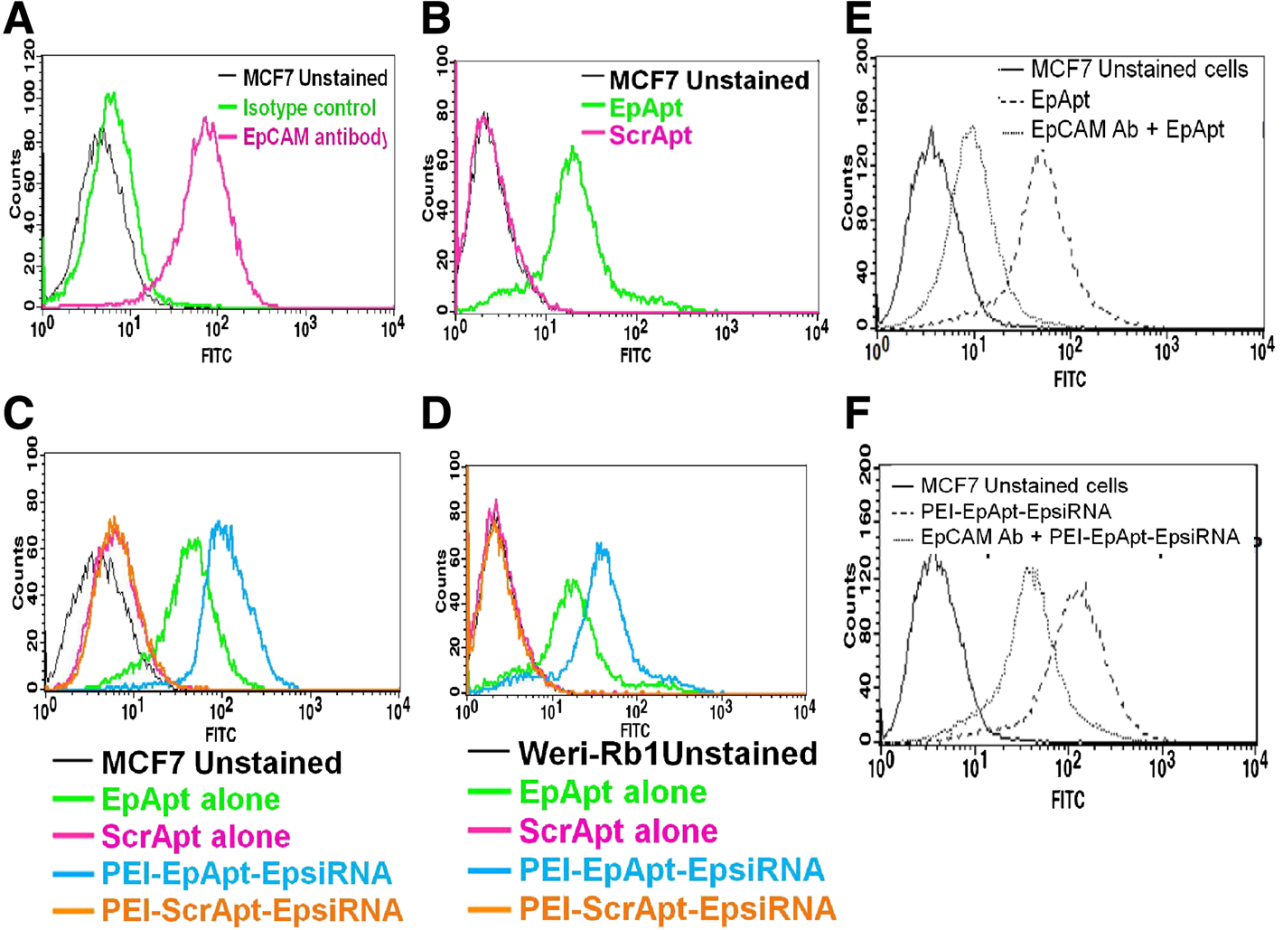
**Expression of EpCAM & binding of the complexes on cells. A**. Histogram overlay plot showing the expression levels of EpCAM protein in MCF-7 cells were evaluated using antibody based method and flow cytometry. **B**. Histogram overlay plot of MCF-7 cells bound to EpApt/EpDT3 or ScrApt/ScrDT3. **C**. Histogram overlay plot of MCF-7 cells bound to aptamer alone or PEI-Apt-siRNA complexes. **D**. Histogram overlay of WERI-Rb1 cells. Aptamer alone or complexes were added to cells and incubated for 2 h, washed & checked by flow cytometry. **E**. Flow cytometry analysis and histogram overlay plot showing the cells blocked with EpCAM antibody before incubating with EpDT3-FI or with EpDT3-FI alone and unstained cells. **F**. Flow cytometry analysis and histogram overlay plot of cells blocked with antibody followed by incubation with PEI-EpApt-SiEp.

**Figure 4:**
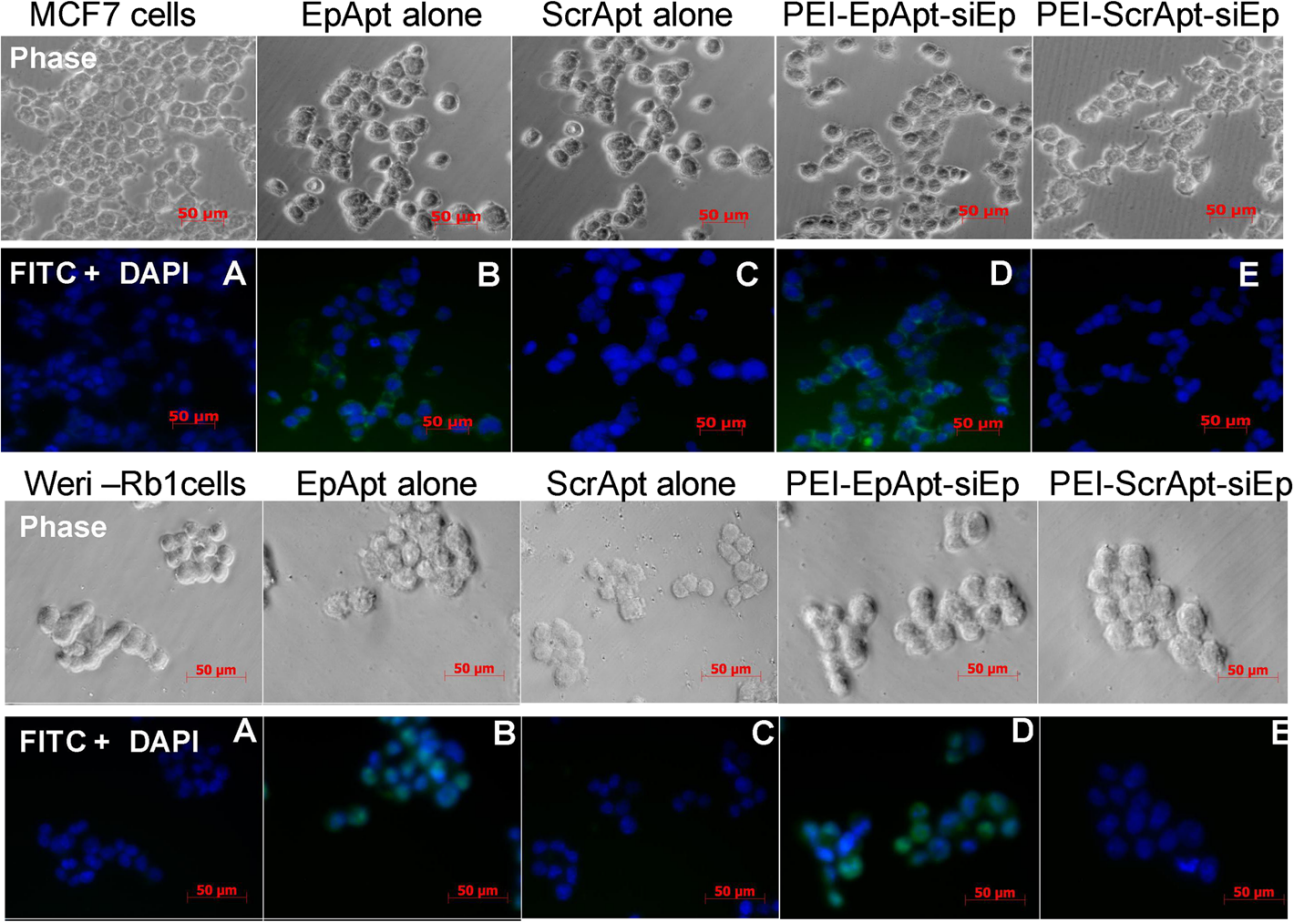
**Cell Uptake of the PEI nanocomplex by MCF-7 and WERI-Rb1 cells.** The fabricated PEI complexes and free aptamer were added to MCF-7 cells (upper panel **A**-**E**), WERI-Rb1 cells (lower panel **A**-**E**) and incubated for their uptake at 37°C for 4 h followed DAPI counterstaining and microscopic evaluation. Images were taken at 40× using AxioObserver fluorescent microscope. Legend on the top of phase image represents the aptamer or nanocomplex added to the respective panels.

The cell binding and uptake of the PEI-EpApt-SiEp complex were studied in MCF-7 and WERI-Rb1 cell lines. Initially, we studied the expression of EpCAM in retinoblastoma and the data generated by us in WERI-Rb1 cell lines is represented in Additional file 2: Figure S1A, [8]. Similarly the expression of EpCAM in MCF-7 has also been studied earlier [2], and the binding of EpCAM aptamer to breast cancer cells, MCF-7 cell line is published [5]. The expression levels of EpCAM proteins in MCF-7 cells are higher compared to the WERI-Rb1 cells (Figure 3A). Similar to the expression levels of the protein, the aptamer binding was higher in MCF-7(Figure 3B). The uptake of aptamer and PEI-Apt-SiEp nanocomplexes was monitored using flow cytometry and the cells bound to PEI-EpApt-SiEp had increased fluorescent intensity compared to the cells bound with EpApt alone in both MCF-7 and WERI-Rb1 cell lines (Figure 3C and D). The ScrApt or PEI-ScrApt-SiEp did not show any binding onto the cell lines. The blocking of the cell surface EpCAM protein by the EpCAM antibody had decreased the binding of EpCAM aptamer alone or PEI-EpApt-SiEp (Figure 3E & F). The cellular uptake of the aptamer alone or the aptamer nanocomplex in MCF-7 and WERI-Rb1 cells was visualized using fluorescent microscopy. The PEI nanocomplex on MCF-7 and WERI-Rb1 cells showed intense membrane staining compared to the EpApt alone (Figure 4). The PEI-EpApt-siRNA nanocomplex exhibited greater binding than EpApt alone (Figure 4B and D). There was no binding when ScrApt or ScrApt-nanocomplex was used in both the cell lines (Figure 4C, E upper panel and 4C, E lower panel). Thus, the specificity of the EpCAM aptamer towards the target is in agreement with the above data.

### Silencing efficiency of the nanocomplex

**Figure 5:**
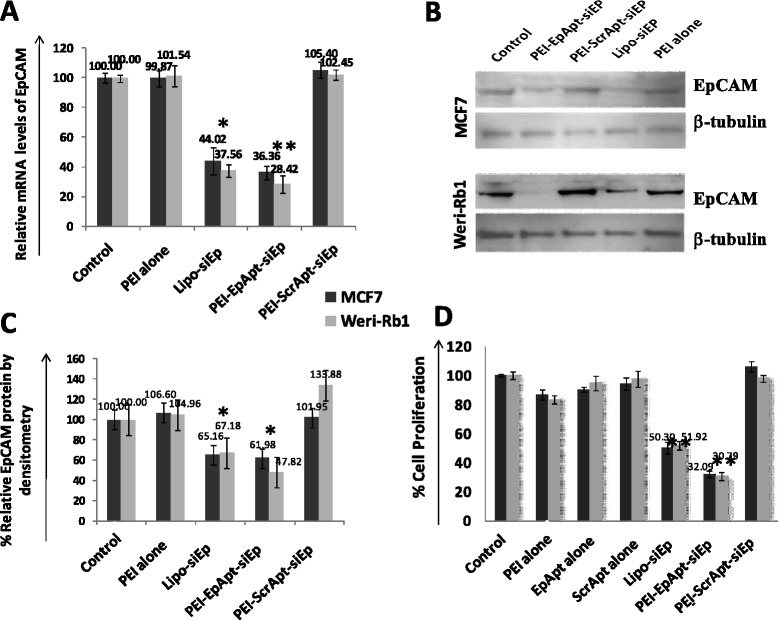
**Expression of EpCAM post silencing using PEI Nanocomplexes. A**. qPCR analysis, graph showing the fold change in the EpCAM expression levels, calculated by normalizing with untreated controls and using GAPDH as internal control gene. qPCR was performed post treatment with PEI alone or PEI-Apt-siRNA complexes or silencing using Lipo-siEp. **B**. Immunoblots were performed for EpCAM and beta-actin to check the silencing efficiency at protein level using EpCAM C-10 primary antibody from Santa Cruz at 1:400 dilution, secondary anti- mouse IgG HRP from Santacruz at 1:1000 dilution. **C**. Densitometry of the immunoblot normalized with tubulin-internal control and untreated control. **D**. Cell proliferation was assessed by performing MTT assay. The graph represents the percentage cell proliferation after treatment with the aptamer alone or PEI alone or PEI-Apt-siRNA complexes. Percentage cell proliferation was calculated considering untreated cells as controls in MCF-7 &WERI-Rb1 cell lines.

The EpCAM silencing by PEI-EpApt-SiEp nanocomplex on both MCF-7 and WERI-Rb1 cells were studied by monitoring the mRNA and protein level using qPCR and Western blotting, respectively. The PEI-Apt-siRNA nanocomplex was effective in silencing the EpCAM compared to the native siRNA transfected using lipofectamine 2000 (Figure 5A). The EpCAM gene was downregulated about 56 and 62% in SiEp treated MCF-7 and WERI-Rb1 cells, while the treatment with PEI-EpApt-SiEp resulted in significant (P value > 0.05) higher levels of downregulation of about 64 and 72% in MCF-7 and WERI-Rb1, respectively. The downregulation was higher in WERI-Rb1 compared to MCF-7 cells. EpCAM silencing was not observed in PEI alone or PEI-ScrApt-SiEp nanocomplex treatments. The EpCAM protein expression,

in PEI alone or PEI-ScrApt-SiEp (showed upregulation only in WERI-Rb1) treated cells did not show significant change compared to untreated cells which are in agreement with mRNA levels (Figure 5B, 3rd and 5th lane). A reduction in EpCAM protein expression was observed in PEI-EpApt-SiEp nanocomplex and siRNA treated cells (Figure 5B, 2nd and 4th lane). The percent reduction in the proteins levels were quantified by densitometry of Western blots. A 35 and 33% reduction in EpCAM expression was observed using lipofectamine mediated transfection of SiEp in MCF-7 and WERI-Rb1 cells, respectively. Furthermore, 38 and 52% reduction in EpCAM levels was obtained with PEI-EpApt-SiEp in MCF-7 and WERI-Rb1 cells, respectively (Figure 5C). There was a positive correlation between the mRNA and protein expression levels in the control, PEI-ScrApt-siEp and PEI-EpApt-SiEp group of MCF-7 and WERI-Rb1 cells (R^2^ value 0.99 and 0.86) (Additional file 2: Figures S2B & C). We performed MTT assay to evaluate the cell proliferation to determine if the changes in EpCAM protein levels had effect on the cell viability. A significant decrease in the cell viability was observed in the cells treated with PEI-EpApt-SiEp nanocomplex compared to siRNA alone (P value < 0.05) (Figure 5D). In MCF-7 and WERI-Rb1 cells, transfection with the SiEp showed 50 and 52% inhibition, respectively whereas the PEI-EpApt-SiEp showed higher inhibition(69% and 68% inhibition, respectively) in these cell lines (P < 0.001). A negligible effect on the cell proliferation was observed in the aptamer alone or PEI alone or PEI-ScrApt-SiEp nanocomplex treated cells. One-way ANOVA analysis showed significant difference (P <0.0005) between the subgroups. Furthermore, to analyze the significant difference between the SiEp treated group and PEI-EpApt-SiEp group, unpaired student t-test was performed and found to have significant difference (P < 0.005) in both the cell lines.

## Discussion and conclusions

There are challenges behind the targeted delivery of siRNA specifically to cancer cells and nonviral carriers such as PEI, poly(lactic-co-glycolic acid) (PLGA), liposomes are found to be better delivery systems [30-32]. The PEI nanocomplex has good transfection efficiency for the siRNA delivery purposes. The cytotoxicity of PEI being an issue, can be managed by using optimal levels that are non-toxic as well have uncompromised efficiency to deliver siRNA/pDNA. Charge stabilization of the PEI is found to be necessary to maintain uniform size [33]. The size and charge of the PEI nanoparticles synthesized in the present study are in agreement with previous reports on PEI nanoparticle fabrications using sgc-8c and PSMA, respectively [34,35]. PEI/pDNA polyplexes assembled with PTK7/sgc-8c aptamer showed particle size ranging from 160–275 nm. In the PSMA aptamer and siRNA chimera assembly on quantum dots surrounded by PEI (QD-PEI), surface charge reportedly drops down upon the siRNA and aptamer addition, which was observed in our study too. A negative charge was observed on the PEI nanocomplex in the presence of siRNA and aptamer compared to the PEI nanocore alone. The hydrodynamic size was higher compared to the size of the particles observed by TEM due to the fact that aptamer and siRNA present on the surface, increased the electron dense nanocore size by 30 nm (hydrodynamic size). The TEM gives the nanocore size and not the surface aptamer and siRNA molecules [36]. The initial influence of the serum proteins on the nanocomplex binding to cells were managed by adding the complex in serum free media, followed by replenishment with serum containing media, to enhance the uptake of the nanocomplex by the cell. The presence of serum in general helps to prevent cytotoxicity of the PEI by increasing membrane integrity of the cell but leads to lower cellular uptake of the particles [37,38].

The scramble nanocomplex had minimal or no binding on cells that proves the specificity of the nanocomplex in this study. The increase in mean fluorescence intensity (MFI) of cells bound with nanocomplex fabricated with EpApt confirms that more than an aptamer is engaged per PEI nanocomplex. Hence binding of nanocomplex to a receptor compared to an aptamer alone results in the increase in MFI. Blocking the EpCAM receptor using a monoclonal antibody reduced the binding of aptamer alone and PEI-EpApt-SiEp nanocomplexes exhibiting lower MFI, confirming the receptor specific binding. The observation that less aptamer is required to saturate the cancer cells in the PEI nanocomplex compared to the EpApt alone i.e., 400 nM of aptamer required to show 90% cell binding in MCF-7 cells compared to 200 nM required in PEI nanocomplex, showed thereby, better performance/affinity of the nanocomplex. This implies that we can bring down the required concentration of aptamer by preparing nanoformulation. Furthermore, the nanocomplex based formulation increases the bioavailability and reactivity of the aptamer to the receptor. Other group had similar observation while using quantum dot-PEI-siRNA-aptamer targeted to PSMA in prostate cancer [34]. The observed marginal binding not mediated by the receptor mediated endocytosis, could be due to the free positively charged PEI groups, which might access the negatively charged cell membrane. Similar non specific binding was observed in the earlier reports [25].

The PEI alone or scramble aptamer targeted complex did not show any effect on the EpCAM gene expression whereas the silencing efficiency of the EpCAM aptamer targeted nanocomplex had reduced EpCAM expression. The EpCAM silencing was considered as a read out for the delivery of the siRNA inside cells, as the aptamer was found to be internalized as observed by microscopy. The targeted gene silencing was better than the conventional lipofection. Other investigators had observed gene silencing effect using PEI nanocomplex targeted against PTK7 and PSMA [29,34]. The mechanism behind the better silencing effect exerted by our nanocomplex is due to the guided delivery of EpCAM siRNA to the EpCAM overexpressing cells. The downregulation of EpCAM mRNA in both the EpCAM overexpressing and moderately expressed cell population thus lead to the cumulative effect. EpCAM antibody-gold nanoparticle based targeting of siEp using PEI had 6 fold enhanced silencing of EpCAM gene expression than the lipofectamine or untargeted siRNA delivery [28]. The fold change observed in the current study is lesser than the antibody mediated siRNA delivery, but the functional activity of our nanocomplex is superior and targeted by EpApt to induce cytotoxicity specifically on EpCAM overexpressing cells. Thus the PEI nanocomplex can be further expanded to the delivery of ribozymes and DNAzyme. Similarly drugs such as doxorubicin were delivered using PEI nanocomplexes [39]; these can be made targeted by tagging aptamers on their surface. In the present study, we fabricated a nanoformulation of PEI with aptamer and siRNA and showed the efficient targeting ability to the EpCAM positive cells. The targeted nanoformulation had better gene silencing activity than the conventional silencing mechanisms. This can be further translated to *in vivo* system for therapeutic purpose.

## Additional files

Additional file 1: **Supplementary materials and methods.**

Additional file 2: Figure S1. Effect of media and serum on PEI nanocomplexes. A. The hydrodynamic diameter of the PEI-Apt-siRNA complexes prepared in medium with and without serum were measured in zetasizer and ploted as histogram overlay plot against the percent number distribution. B. Graphs showing the zeta potential of the complexes prepared in medium with and without serum. **Figure S2.** EpCAM expression in WERI-Rb1 cell line. A. The histogram overlay plot shows the expression level of EpCAM by flow cytometry assay. The isotype control vs the EpCAM expression reveals (blue) about 35% positive cells for the expression. (Figure represented from earlier publication) [8]. The mRNA and protein levels of EpCAM across the control, PEI-ScrApt-SiEp and PEI-EpApt-siRNA were compared by fixing the protein levels in x-axis and mRNA levels in y-Axis. The R2value is determined from the trend line drawn between the samples. The equation is displayed on the left for both the cell lines MCF-7 (B) and WERI-Rb1 (C).

## Abbreviations

EpCAM: Epithelial cell adhesion molecule
PEI: Polyethyleneimine
EpApt: EpCAM aptamer
ScrApt: Scramble aptamer
siEp: siRNA targeting EpCAM
