# Correction to: EpCAM aptamer mediated cancer cell specific delivery of EpCAM siRNA using polymeric nanocomplex

**DOI:** 10.1186/s12929-020-00687-2

**Published:** 2020-11-08

**Authors:** Nithya Subramanian, Jagat R. Kanwar, Prasanna kumar Athalya, Narayanan Janakiraman, Vikas Khetan, Rupinder K. Kanwar, Sailaja Eluchuri, Subramanian Krishnakumar

**Affiliations:** 1 Department of Nanobiotechnology, Vision Research Foundation, Kamalnayan Bajaj Institute for Research in Vision and Ophthalmology, Tamil Nadu, 18 College Road, Chennai, 600006 India; 2 Nanomedicine Laboratory of Immunology and Molecular Biomedical Research (LIMBR), School of Medicine (SoM), Molecular and Medical Research (MMR) Strategic Research Centre, Faculty of Health, Deakin University, Geelong, VIC 3217 Australia; 3 L & T Ocular Pathology Department, Vision Research Foundation, Kamalnayan Bajaj Institute for Research in Vision and Ophthalmology, Chennai, India; 4 Departments of Ocular Oncology and Vitreoretina, Medical Research Foundation, Sankara Nethralaya, Chennai, India

## Correction to: J Biomed Sci (2015) 22:4 https://doi.org/10.1186/s12929-014-0108-9

After published the article [1] the author became aware of a mistake in the Fig. 4 (upper panel section C). Due to an error that occurred inadvertently at the time of figure assembly, an incorrect image for ScrApt alone sample was included.

The correct version of the Fig. 4 is given below. This change does not affect the conclusions and interpretations of the article in any way. The authors sincerely apologize for this unintentional error.

**Fig. 4:**
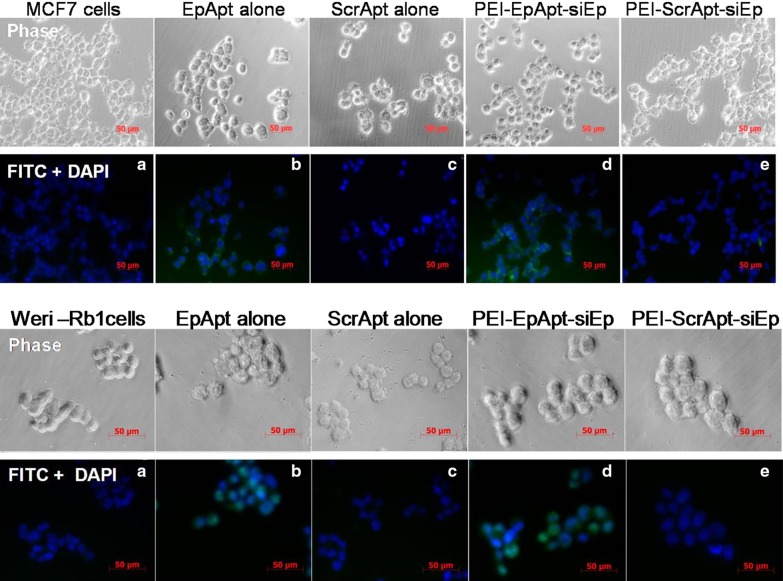
Cell Uptake of the PEI nanocomplex by MCF-7 and WERI-Rb1 cells. The fabricated PEI complexes and free aptamer were added to MCF-7 cells (upper panel **a**–**e**), WERI-Rb1 cells (lower panel **a**–**e**) and incubated for their uptake at 37 °C for 4 h followed by DAPI counterstaining and microscopic evaluation. Images were taken at 40 × using AxioObserver fluorescent microscope. Legend on the top of phase image represents the aptamer or nanocomplex added to the respective panel
