# Supplementary material for: EpCAM aptamer mediated cancer cell specific delivery of EpCAM siRNA using polymeric nanocomplex
Source: J Biomed Sci. 2015 Jan 9;22:4. doi: 10.1186/s12929-014-0108-9 (PMC4307906; doi:10.1186/s12929-014-0108-9)
Supplement: Supplementary file 1 — Supplementary materials and methods. [file 12929_2014_108_MOESM1_ESM.pdf]

Supplementary materials and methods

Materials:

Branched Polyethyleneimine polymer (60 kDa, catalog #P3143) and sodium citrate (catalog #C8532) were purchased from Sigma Aldrich (Bangalore, India). EpCAM aptamer (EpApt) with 2'F pyrimidines and scramble aptamer (ScrApt) with 2'OMe pyrimidines carrying the sequence (5′GCGACUGGUUACCCGGUCG-3′) and SYBR green master mix (Finnzymes) was obtained from thermo fisher scientific (Lafayette, CO). Both EpCAM and scramble aptamer were fluorescein-labeled (FI), siRNA against EpCAM (SiEp) targeting the transcript region 5'-GGAACUAAUGCUAACUATT-3' were purchased from Qiagen (Mainz, Germany). QPCR primers were obtained from Sigma-Aldrich (Bangalore, India). TEM was performed for the PEI-Apt-siRNA nanocomplex (200nM EpApt and 200nM siRNA) imaged at 80V (TEM, Philips, CM12 STEM, Netherlands).

Western blotting:

The immunoblot analysis was performed to check the EpCAM protein expression in both cell lines treated with the PEI-Apt-siRNA nanocomplexes or lipofectamine-SiEp. Briefly, after 48hours of the treatment, the cells were washed twice with 1XPBS and lysed with (Radio Immuno Precipitation Assay) RIPA buffer, equal concentration of protein was separated on 12% SDS-PAGE gel, transferred on to nitrocellulose membrane (HybondEcl, Amersham, GE Biosciences). The EpCAM (C-10) antibody (Santa cruz Bio., Texas, USA) was used at a dilution of 1:200 and anti-tubulin antibody at 1:400 dilution (Santa cruz Bio., Texas, USA) followed by the addition of the anti-mouse HRP antibody (Sigma Aldrich, Bangalore, India) at 1:3000 dilution. Chemiluminescent based detection was performed using Western maximum signal sensitivity kit (Pierce) by autoradiography and Fluorchem FC3 (Protein simple). Densitometry analysis was performed on the replicate blots using imageJ software and percentage expression is calculated and plotted.

Cellular uptake of the aptamer and nanocomplex:

The unstained cells and the scrambled aptamer added cells served as controls. The uptake of the aptamer alone and PEI-Apt-siRNA nanocomplex were visualized using fluorescent Axio Observer microscopy (Zeiss, Germany). Briefly, MCF7 and WERI-Rb1 cells were seeded at a

density of 20,000 per well in 24 well plate. The aptamer alone or PEI-Apt-siRNA nanocomplex was added at a reaction volume of 250µl after 24hrs. The cells were incubated with complexes for 4hrs, washed two times with 1XPBS followed by fixing with 4% paraformaldehyde and nuclear staining using DAPI and imaging.

Cytotoxicity of PEI aptamer-siRNA nanocomplex on cell culture:

The effect of nanocomplexes on cell proliferation was studied in MCF7 and WERI-Rb1 cells. Cells were seeded at a density of (60% confluency). 24hrs after seeding the PEI (0.3µg/ml) PEI-Apt-siRNA nanocomplexes or lipofectamine-siRNA prepared in serum free media were added followed by addition of complete media to the cells. The cells were further incubated for 48 hours, followed by MTT assay (MTT, Sigma Aldrich, Bangalore, India).

MTT assay:

The effect of the PEI nanocore or nanocomplex was tested in MCF7 and WERI-Rb1 cells using MTT assay. 48hrs post-treatment cells were replenished with 100µl of media containing 10µl of MTT (5mg/ml) and incubated for 4hrs at 37°C. Then media was removed and the MTT crystals formed were dissolved using 100µl of DMSO per well and the absorbance was measured at 570nm. All experiments were performed in triplicate.

Supplementary Figure legends:

Supplementary Figure 1. Effect of media and serum on PEI nanocomplexes. A. The hydrodynamic diameter of the PEI-Apt-siRNA complexes prepared in medium with and without serum were measured in zetasizer and ploted as histogram overlay plot against the percent number distribution.**B.** Graphs showing the zeta potential of the complexes prepared in medium with and without serum.

Supplementary Figure 2. EpCAM expression in WERI-Rb1 cell line. A. The histogram overlay plot shows the expression level of EpCAM by flow cytometry assay. The isotype control vs the EpCAM expression reveals (blue) about 35% positive cells for the expression. (Figure re-presented from earlier publication)[8] **EpCAM knockdown correlation.** The mRNA and protein levels of EpCAM across the control, PEI-ScrApt-SiEp and PEI-EpApt-siRNA were compared by fixing the protein levels in x-axis and mRNA levels in y-Axis. The R^2value is

determined from the trend line drawn between the samples. The equation is displayed on the left for both the cell lines MCF-7 (**B**) and WERI-Rb1 (**C**).

Supplementary Figure legends

Supplementary Figure 1. Effect of media and serum on PEI nanocomplexes. A. The hydrodynamic diameter of the PEI-Apt-siRNA complexes prepared in medium with and without serum were measured in zetasizer and ploted as histogram overlay plot against the percent number distribution.**B.** Graphs showing the zeta potential of the complexes prepared in medium with and without serum.

Supplementary Figure 2. EpCAM expression in WERI-Rb1 cell line. A. The histogram overlay plot shows the expression level of EpCAM by flow cytometry assay. The isotype control vs the EpCAM expression reveals (blue) about 35% positive cells for the expression. (Figure re-presented from earlier publication)[8]. The mRNA and protein levels of EpCAM across the control, PEI-ScrApt-SiEp and PEI-EpApt-siRNA were compared by fixing the protein levels in x-axis and mRNA levels in y-Axis. The R^2value is determined from the trend line drawn between the samples. The equation is displayed on the left for both the cell lines MCF-7 (**B**) and WERI-Rb1 (**C**).
