# Supplementary figures and images for: EpCAM aptamer mediated cancer cell specific delivery of EpCAM siRNA using polymeric nanocomplex
Source: J Biomed Sci. 2015 Jan 9;22:4. doi: 10.1186/s12929-014-0108-9 (PMC4307906; doi:10.1186/s12929-014-0108-9)

## Supplementary Figure 1

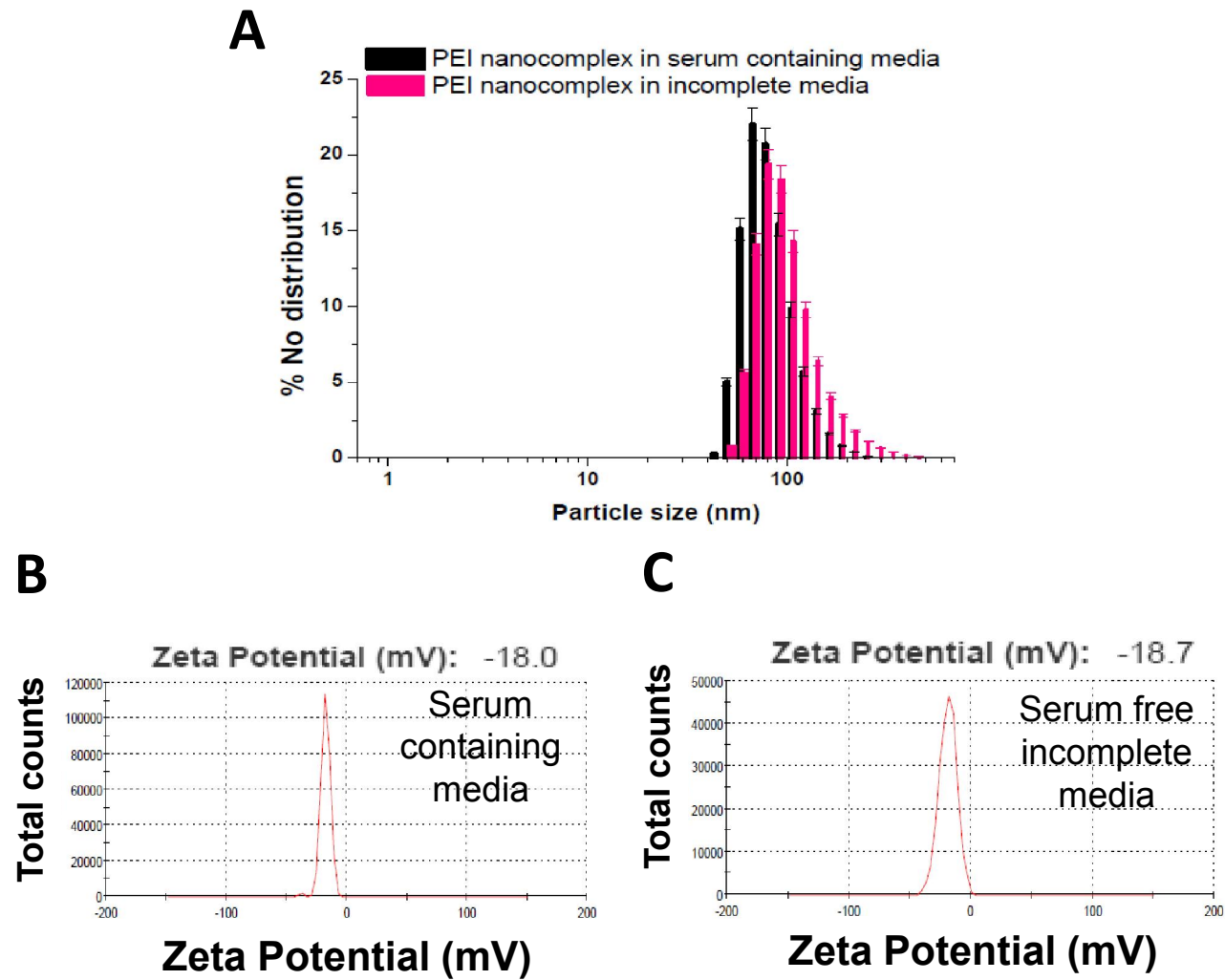

## Supplementary Figure 2

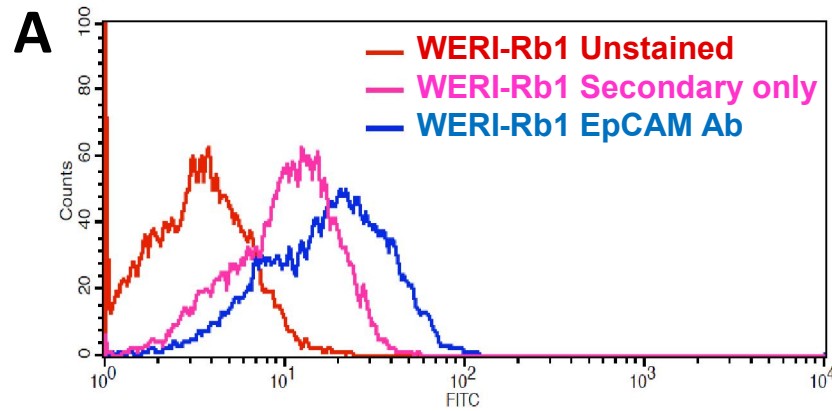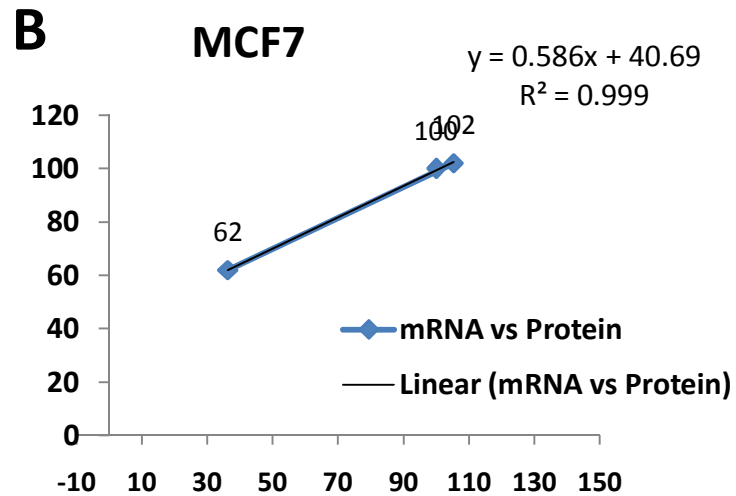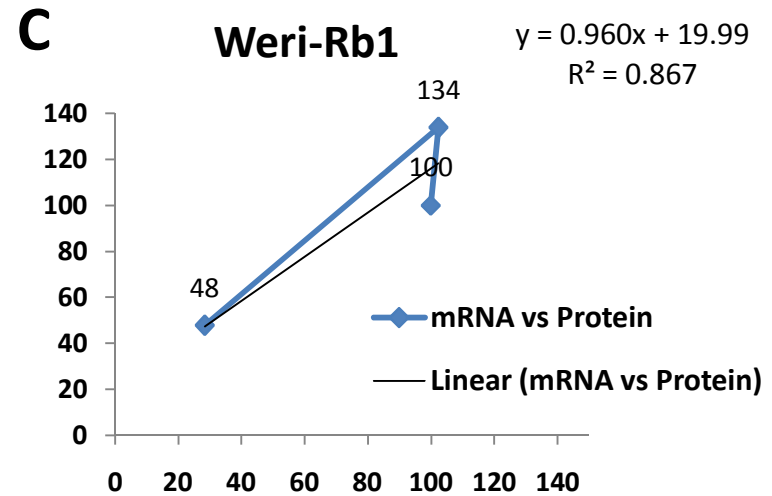

Supplement: Supplementary file 2 — Effect of media and serum on PEI nanocomplexes. A. The hydrodynamic diameter of the PEI-Apt-siRNA complexes prepared in medium with and without serum were measured in zetasizer and ploted as histogram overlay plot against the percent number distribution. B. Graphs showing the zeta potential of the complexes prepared in medium with and without serum. Figure S2. EpCAM expression in WERI-Rb1 cell line. A. The histogram overlay plot shows the expression level of EpCAM by flow cytometry assay. The isotype control vs the EpCAM expression reveals (blue) about 35% positive cells for the expression. (Figure represented from earlier publication) [8]. The mRNA and protein levels of EpCAM across the control, PEI-ScrApt-SiEp and PEI-EpApt-siRNA were compared by fixing the protein levels in x-axis and mRNA levels in y-Axis. The R2value is determined from the trend line drawn between the samples. The equation is displayed on the left for both the cell lines MCF-7 (B) and WERI-Rb1 (C). [file 12929_2014_108_MOESM2_ESM.pdf]
